# Temporal trends and spatial patterns of Hepatitis C-related mortality in Brazil

**DOI:** 10.11606/s1518-8787.2025059006139

**Published:** 2025-03-24

**Authors:** Elodie Bomfim Hyppolito, Alberto Novaes Ramos, Anderson Fuentes Ferreira, Thor Oliveira Dantas, José Milton de Castro Lima, Taynara Laís Silva, Roberto da Justa Pires

**Affiliations:** I Universidade Federal do Ceará. Faculdade de Medicina. Programa de Pós-graduação em Saúde Pública. Fortaleza, CE, Brasil; II Secretaria de Saúde do Estado do Ceará. Hospital São José de Doenças Infecciosas. Fortaleza, CE, Brasil; III Universidade de Fortaleza. Fortaleza, CE, Brasil; IV Universidade Federal do Acre. Centro de Ciências da Saúde e do Desporto. Rio Branco, AC, Brasil; V Universidade Federal do Ceará. Faculdade de Medicina. Departamento de Clínica Médica. Fortaleza, CE, Brasil; VI Universidade Federal do Ceará. Faculdade de Medicina. Departamento de Saúde Comunitária. Fortaleza, CE, Brasil

**Keywords:** Hepatitis C, Mortality, Spatial Analysis, Time Series Study, Epidemiology

## Abstract

To analyze the trends and spatial patterns of hepatitis C-related mortality in Brazil from 2000 to 2020.

A population-based, mixed ecological study of spatial and temporal trends, using secondary data from death certificate (DC) registries, in which hepatitis C was mentioned as an underlying or associated cause of mortality. Temporal trends were analyzed by joinpoint regression, and spatial analysis by the distribution of adjusted rates by age and sex, and spatial autocorrelation by the local Moran index and the Getis-Ord Gi* index (Gi star).

From 2000 to 2020, 64,029 deaths due to hepatitis C were recorded in the Mortality Information System (SIM), representing 0.26% of deaths in Brazil. Most deaths were due to underlying causes (n = 33,652, 52.6%). Areas with high rates in all five analyzed periods were identified in the states of São Paulo, southern Minas Gerais, southern Rio de Janeiro, northern Paraná, southern and coastal Santa Catarina, eastern Mato Grosso do Sul, and Rio Grande do Sul. The states of Acre and southern Amazonas showed high rates after 2004, which spread to northern Rondônia from 2016 to 2020. The joinpoint regression model showed an increasing trend in hepatitis C mortality in Brazil from 2000 to 2015, but a decreasing trend from 2016-2020. The mortality rate was higher in men and people over sixty years of age.

Differences were observed in the temporal and spatial trend of hepatitis C mortality in different regions of Brazil. These data may support the design of hepatitis C elimination strategies in Brazil, according to regional specificities.

## INTRODUCTION

Hepatitis C (HC) is a worldwide infection caused by an RNA virus that is primarily transmitted through blood. Patients who received blood before 1993, intravenous drug users, healthcare professionals, and adults over 45 years of age are the populations with the highest prevalence of this infection^1 , 2^.

A recent serological survey, conducted from 2005 to 2009, showed an overall prevalence of anti-HCV antibodies of 1.38% in the capital cities of Brazil. Seropositivity ranged from 0.68% in the Northeast Region to 2.10% in the North Region^1^. Most will remain infected as chronic carriers. If left untreated, chronic hepatitis C virus may lead to cirrhosis in up to one third of those infected individuals after 20 to 30 years. Hepatocarcinoma occurs in 1% to 4% of patients with cirrhosis. Hepatitis C is the most commonly reported hepatitis in Brazil, accounting for 38.1% of all viral hepatitis cases reported in the country. In 2020, 47.9% of these cases occurred in the Southeast region, 35.2% in the South, 5.9% in the Northeast, 5.6% in the Central-West, and 5.4% in the North. Hepatitis C is also the most lethal form of viral hepatitis in Brazil, being the third cause of hepatocarcinoma after hepatitis B and alcohol, and the second cause of liver transplantation ^3 , 4^.

Since 2015, the Unified Health System has started to treat hepatitis C with drugs of direct antiviral action (DAAs) which, despite their high cost, cure most of the patients. The researchers reviewed multicenter trials involving 3,939 patients across the country. Overall, Standard Variable Rates were greater than 95%^5^.

Few studies have evaluated mortality from hepatitis C in Brazil. One study showed that from January 2008 to December 2014 a total of 8,106,219 deaths were registered in Brazil. During this period, 0.43% (n = 34,978) of deaths had International Classification of Diseases (ICD-10) codes related to viral hepatitis as the primary, secondary, or contributing cause of death^6^. In another study evaluating the period from 2008 to 2018, approximately 140,000 newly diagnosed cases of hepatitis C were considered anti-HCV and HCV RNA positive, with an increasing trend in Brazil, but a decreasing trend in the mortality rate during the period analysed^7^. Another study addressed mortality from hepatitis A, B, and C in Brazil, from 2001 to 2020, and found similar results^8^.

This study aims to analyze the burden of hepatitis C mortality, temporal trends, and spatial patterns of hepatitis C mortality in Brazil, from 2000 to 2020.

## METHODS

### Study Design

We conducted a mixed ecological study to analyze the spatial distribution and temporal trends. We used information from selected Death Certificates (DC) with hepatitis-related deaths (associated and underlying causes), in Brazil, according to the International Statistical Classification of Diseases and Health Problems in its tenth revision (ICD-10). The following ICD-10 codes were used for identification: B17.1 – Acute hepatitis C; B18.2 – Chronic viral hepatitis C.

### Study Area

The study was conducted in Brazil, the largest country in South America in terms of area and population, with an area of 8.5 million km² and an estimated population of 213.3 million inhabitants. The Southeast region has the highest population density (86.9 inhabitants per km²), while the North region has the lowest (4.1 inhabitants per km^2^)^9^.

According to data from the 2010 Brazilian census, Brazil has a Gini index of 0.60, reflecting the inequality in the country, which is more pronounced in the North and Northeast regions (Gini of 0.62)^10^.

The 2019 National Continuous Household Sample Survey (PNAD) had the highest population percentages in the age groups from 25–39 (23.1%) and 40–59 (26.2%) age groups. The South (17.4%) and Southeast (17.1%) regions had the highest proportions of older population (60 years and over)^11^.

### Data Sources

Information was obtained from the Brazilian Mortality Information System (SIM – *Sistema de Informação sobre Mortalidade*), which was developed by the Ministry of Health to analyze mortality data in the country and is based on analyzed and consolidated DCs. Medical professionals are responsible for issuing the Death Certificates, filling in all the fields, and signing at the end. In this study, we considered all deaths in which hepatitis C was listed as either as the underlying cause or as an associated cause of death, from 2008 to 2020^12^.

Data published in the 2023 Viral Hepatitis Epidemiological Bulletin were also included^3^. Confirmed cases of hepatitis C were considered as follows: individuals with both reactive serological markers (anti-HCV and HCV RNA) from 2000 to 2014, and individuals testing positive for either marker (anti-HCV or HCV RNA) from 2015 to 2023. The change in the reporting criteria was adopted by the Brazilian Ministry of Health with the aim of increasing the sensitivity of detection of new cases of hepatitis C in the national territory. Despite the change, the Brazilian Ministry of Health database provides data on both confirmation criteria for the entire study period. Therefore, this study present data for both criteria from 2000 to 2023^13^.

### Variables, Statistical Analysis

The following variables were analyzed: sex/gender (male, female), age group (< 15, 15–29, 30–39, 40–49, 50–59, 60–69, and ≥ 70 years), race/skin color (White, Black [Afro-Brazilian/Afro-descendant], Yellow [Asian-descendant], *Pardo* [Brown/Mixed race], and Indigenous [Amerindians]), region of residence (North, Northeast, Southeast, South, and Central-West regions), and residence in a state capital city (yes/no). Relative frequencies were calculated using the direct standardization method, including crude mortality rates (mean deaths/population) and sex- and age-adjusted rates, and with the 2010 Brazilian population as a reference per 100,000 inhabitants. Differences between groups were analyzed using the relative risk (RR) estimated with the corresponding respective confidence intervals (95%CI). Pearson’s chi-squared test (χ^2^) was used to determine statistically significant differences between the groups^14^.

### Trend Analysis

Temporal trends in hepatitis C-specific mortality rates were calculated using joinpoint regression (Joinpoint Regression Program, version 4.4.2; Statistical Research and Applications Branch, National Cancer Institute, available at:  https://surveillance.cancer.gov/joinpoint/) ^15^ 15). This statistical method uses annual trend tests to determine the fit of a series of lines and their inflection points on a logarithmic scale. In addition, we used the Monte Carlo permutation method as a statistical significance test to obtain the best line for each segment. We tested and validated the annual percent change (APC) and the average annual percent change (AAPC), with respective 95%CIs to identify increase (positive APC/AAPC), decrease (negative APC/AAPC), or no trend (APC/AAPC without statistical significance)^16^.

### Spatial Analysis

Four different time series were used for the spatial analysis: 2000–2003, 2004–2007, 2008–2011, 2012–2015, 2016–2020. The locations included refer to the municipality of residence. The technique that analyzed the spatial distribution of deaths took into account the standardized rates by age and sex, as well as the deaths of neighboring municipalities per 100,000 inhabitants. Thematic maps were produced to show whether the distribution patterns remained similar and how areas with higher rates changed over time. The age groups used to calculate the mortality rates were standardized as 0–14, 15–29, 30–39, 40–49, 50–59, 60–69, and ≥ 70 years. The standardized rates (age and sex) were calculated using the direct standardization method, with the population of Brazil in 2010 as reference^17 , 18^.

Spatial dependence was assessed using the Getis-Ord indices of {G} and {Gi*} (Gi star). It was assumed that a high value of score “Z” and a low value of “p” of a parameter indicates a spatial agglomeration of high values. Conversely, a low negative “Z” score and a small “p” value indicate spatial clusters of low values. These analysis parameters identified the presence of high or low value aggregates in groups of municipalities, allowing the identification of high and low risk clusters on the map.

The presence of local autocorrelation was checked using the Local Moran’s Index. The method is based on the use of local indicators of spatial association. The results show the municipalities around which there is a significant clustering of similar values. Statistical analyses were performed using the Stata software (version 11.2; StataCorp LP, College Station, TX, USA), and ArcGIS software (version 9.3; Environmental Systems Research Institute, Redlands, CA, USA) to estimate autocorrelation indicators and produce thematic maps^19^.

### Ethical Considerations

The project was approved by the Research Ethics Committee of the Hospital São José de Doenças Infecciosas (HSJ), number 4.149.901, CAAE: 53791616.7.2007.5044. This research only uses public secondary data, available through free access to official websites of the Ministry of Health and with anonymized identification data of hospitalizations and/or deaths. Therefore, the principles of Resolution no. 466 of December 2012, of the National Health Council of Brazil, have been followed for research involving human subjects.

## RESULTS

**Figure 1.**
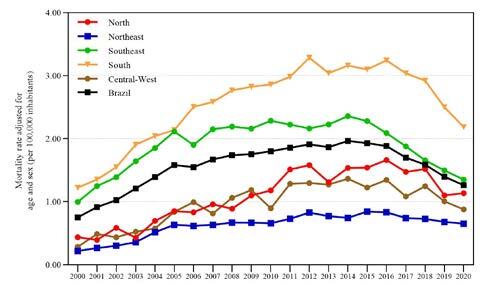
Age and sex-adjusted Hepatitis C-related rates (per 100,000 inhabitants), by Brazilian regions, 2000–2020.

From 2000 to 2020, 64,029 deaths occurred due to hepatitis C, representing 0.26% of deaths in Brazil (n = 24,215,831). Most deaths in the country were due to underlying causes (n = 33,652, 52.6%). Acute hepatitis C was responsible for 13,225 (20.7%) deaths, while chronic viral hepatitis C was identified in 50,821 (79.4%) deaths. Both forms were found together in 17 DCs. The average adjusted rate was 1.57/100,000 inhabitants (95%CI 1.56–1.58) (Table 1). The adjusted mortality rates over time showed a pattern of increase until 2012, stabilization by 2016, and a decrease in 2017–2020. The highest averages rates were observed in the South (2.77/100,000 inhabitants), followed by the Southeast (2.09/100,000 inhabitants) region (Figure 1).

Males predominated (n = 41,047, 64.1%, 2.05/100,000 inhabitants [95%CI 2.03–2.07]), aged between 50–59 years (n = 18,711, 29 years), with higher adjusted mortality rates for the age groups 60–69 years (6.69/100,000 inhabitants [95%CI 6.59–6.80]) and 70 years and older (6.88/100,000 inhabitants [95%CI 6.76–7.00]), of white race/skin color (n = 42,178, 65.9%, 1.98/100,000 inhabitants [95%CI 1.89–2.07]), resident in the Southeast region (n = 35,553, 55.5%), with the highest adjusted mortality rates in the South region (2.56/100,000 inhabitants [95%CI 2.52–2.60]) and non-residents in the state capital (n = 36,972, 57.7%), in addition to higher adjusted mortality rates for capital residents (2.76/100,000 inhabitants [95%CI 2.73–2.79]) (Table 1).

**Table 1.** Number and percentage, crude rate (per 100,000 inhabitants), adjusted rate, relative risk, and temporal trend according to Joinpoint regression of Hepatitis C-related mortality by sociodemographic variables, Brazil, 2000–2020.

Indicator/Characteristic	Death	Crude rate	Adjusted rate (95%CI)	Trend		Entire period
	n (%)			Period	APC (95%CI)	AAPC (95%CI)
**Brazil – Total**	64,029 (100.0)	1.58	1.57 (1.56–1.58)	2000–2005	15.1* (12.8 to 17.5)	1.8* (0.1 to 3.5)
				2005–2012	2.8* (1.6 to 4.0)	
				2012–2016	0.2 (-2.9 to 3.4)	
				2016–2020	-10.4* (-12.4 to -8.4)	
**Sex/Gender**						
Male	41,047 (64.1)	2.06	2.05 (2.03–2.07)	2000–2005	15.9* (13.5 to 18.4)	1.8* (0.2 to 3.5)
				2005–2015	2.1* (1.5 to 2.8)	
				2015–2020	-8.2* (-9.6 to -6.7)	
Female	22,975 (35.9)	1.11	1.11 (1.09–1.12)	2000–2005	13.8* (10.1 to 17.6)	1.9* (0.1 to 3.7)
				2005–2011	3.9* (1.4 to 6.5)	
				2011–2016	0.5 (-2.6 to 3.7)	
				2016–2020	-11.9* (-15 to -8.6)	
*Missing values*	7 (0.0)					
**Age group (years)**						
<15	44 (0.1)	0.00	0.00 (0.00–0.01)	2000–2020	-4.3 (-9.6 to 1.4)	-4.3 (-9.6 to 1.4)
15–29	705 1.1)	0.07	0.07 (0.06–0.07)	2000–2005	2.1 (-5.4 to 10.3)	-6.5* (-8.0 to -5.0)
				2005–2020	-8.7* (-10.5 to -6.8)	
30–39	3,627 (5.7)	0.59	0.57 (0.55–0.59)	2000–2005	10.3* (2.8 to 18.3)	-6.3* (-8.3 to -4.3)
				2005–2020	-9.9* (-11.3 to -8.4)	
40–49	10,462 (16.3)	2.02	2.01 (1.97–2.05)	2000–2005	13.1* (10.8 to 15.4)	-2.6* (-4.6 to -0.5)
				2005–2008	0.1 (-6.7 to 7.3)	
				2008–2015	-4.4* (-5.6 to -3.2)	
				2015–2020	-14.0* (-15.8 to -12.2)	
50–59	18,711 (29.2)	4.82	4.81 (4.74–4.88)	2000–2005	16.7* (12.7 to 20.8)	0.1 (-1.9 to 2.2)
				2005–2014	1.4* (0.2 to 2.5)	
				2014–2017	-8.0 (-16.6 to 1.4)	
				2017–2020	-13.8* (-18.7 to -8.7)	
60–69	16,733 (26.1)	6.92	6.69 (6.59–6.80)	2000–2004	15.7* (6.9 to 25.2)	1.6 (-0.1 to 3.4)
				2004–2015	3.6* (2.3 to 5.0)	
				2015–2020	-9.7* (-12.7 to -6.6)	
≥70	13,729 (21.4)	6.97	6.88 (6.76–7.00)	2000–2002	26.5* (3.8 to 54.3)	0.7 (-0.8 to 2.3)
				2002–2007	6.1* (1.3 to 11.1)	
				2007–2016	0.9 (-0.4 to 2.2)	
				2016–2020	-11.0* (-14.2 to -7.7)	
*Missing values*	18 (0.0)					
**Race/color**						
White	42,178 (65.9)	2.25	1.98 (1.89–2.07)	2000–2004	19.1* (14.4 to 24.0)	1.8* (0.0 to 3.6)
				2004–2012	3.1* (1.8 to 4.4)	
				2012–2016	0.1 (-4.4 to 4.7)	
				2016–2020	-11.9* (-14.8 to -8.9)	
Black (Afro-Brazilian/Afro-descendant)	4,513 (7.0)	1.49	1.32 (1.14–1.50)	2000–2006	17.5* (9.0 to 26.6)	2.1* (0.1 to 4.1)
				2006–2016	1.7 (-0.9 to 4.3)	
				2016–2020	-9.4* (-16.7 to -1.4)	
Yellow (Asian-descendant)	442 (0.7)	1.02	0.66 (0.33–0.98)	2000–2002	-46 (-72.3 to 5.1)	-11.1* (-13.1 to -9.1)
				2002–2007	4.1 (-16.5 to 29.8)	
				2007–2020	-12.9* (-16.1 to -9.5)	
*Pardo* (Brown /Mixed race)	13,979 (21.8)	0.80	0.93 (0.86–1.00)	2000–2005	23.1* (17.3 to 29.2)	3.6* (1.6 to 5.6)
				2005–2015	4.7* (3.5 to 5.9)	
				2015–2020	-7.2* (-9.5 to -4.8)	
Indigenous (Amerindians)	54 (0.1)	0.30	-	2000–2020	2.4 (-1.3 to 6.1)	2.4 (-1.3 to 6.1)
*Missing values*	2,863 (4.5)					
**Region of residence**						
North	2,671 (4.2)	0.78	1.10 (1.06–1.15)	2000–2012	11.6* (8.4 to 14.9)	4.8* (2.6 to 7.0)
				2012–2020	-3.2 (-6.8 to 0.5)	
Northeast	6,433 (10.0)	0.56	0.63 (0.61–0.64)	2000–2005	23.5* (17.2 to 30.2)	3.3* (1.5 to 5.2)
				2005–2016	2.8* (1.6 to 4.1)	
				2016–2020	-7.6* (-12 to -2.9)	
Southeast	35,553 (55.5)	2.09	1.90 (1.88–1.92)	2000–2005	14.5* (11.2 to 17.9)	0.8 (-1.0 to 2.5)
				2005–2015	1.1* (0.2 to 2.1)	
				2015–2020	-10.5* (-12.7 to -8.3)	
South	16,656 (26.0)	2.86	2.56 (2.52–2.60)	2000–2006	12.5* (9.8 to 15.2)	2.9* (1.3 to 4.5)
				2006–2012	4.3* (1.9 to 6.8)	
				2012–2018	-0.7 (-2.8 to 1.5)	
				2018–2020	-17.4* (-26.1 to -7.8)	
Central-West	2,716 (4.2)	0.90	0.98 (0.94–1.01)	2000–2012	10.8* (7.2 to 14.4)	3.8* (1.5 to 6.1)
				2012–2020	-4.6* (-8.5 to -0.5)	
*Missing values*	0 (0.0)					
**Residence in a state capital city**						
No	36,972 (57.7)	1.19	1.19 (1.18–1.21)	2000–2005	17.6* (14.6 to 20.6)	2.5* (0.7 to 4.3)
				2005–2015	3.1* (2.3 to 3.8)	
				2015–2020	-8.1* (-9.8 to -6.4)	
Yes	27,057 (42.3)	2.81	2.76 (2.73–2.79)	2000–2005	13.3* (10.9 to 15.8)	0.9 (-0.7 to 2.6)
				2005–2016	1.1* (0.5 to 1.6)	
				2016–2020	-12.9* (-15.1 to -10.7)	
*Missing values*	0 (0.0)					

-: not calculated; *: significantly different from 0 (p < 0.05); N: Number, %: percentage; APC: Annual Percent Change; AAPC: Average Annual Percent Change

### Time Trend

The time trend by the joinpoint analysis showed a total of three (3) breaks for most of the variables analyzed, as presented for the general trend in Brazil, with an increase in the time trend in the period 2000–2005 (APC 15.1, 95%CI 12.8 to 17.5), a further increase in the period 2005–2012 (APC 2.8, 95%CI 1.6 to 4.0) and a decrease in the period 2016–2020 (APC -10.4, 95%CI -12.4 to -8.4). For the sexes, there was an increasing trend in the general period (males: AAPC 1.8, 95%CI 0.2 to 3.5; females: AAPC 1.9, 95%CI 0.1 to 3.7). For age groups, a general downward trend was observed between those aged 15–49 years old (Table 1).

For race/skin color, general upward trends were observed for White (AAPC 1.8, 95%CI 0.0 to 3.6), Black (AAPC 2.1, 95%CI 0.1 to 4.1), and *Pardo* (AAPC 3.6, 95%CI 1.6 to 5.6). Regionally, the strongest general upward trends were observed in the North (AAPC 4.8, 95%CI 2.6 to 7.0) and the Central-West (AAPC 3.8, 95%CI 1.5 to 6.1) regions. An upward trend was identified in mortality rate among non-residents of capital cities (AAPC 2.5, 95%CI 0.7 to 4.3) (Table 1).

### Spatial Distribution and Spatial Analysis

**Figure 2.**
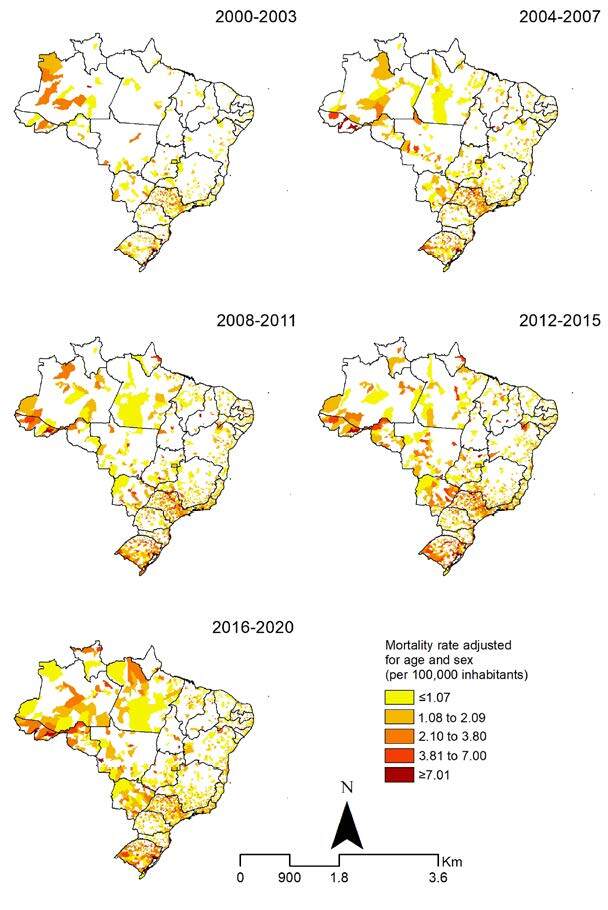
Spatial distribution of rate adjusted for age and sex of Hepatitis C-related mortality rates (per 100,000 inhabitants) by the municipality of residence in Brazil, 2000–2020

The spatial distribution of adjusted rates showed a heterogeneous pattern, with concentrations of high rates in the states of São Paulo, Rio de Janeiro, and Rio Grande do Sul. The states of Acre, Pará, Rondônia, and Mato Grosso do Sul showed an increase in areas with high rates after the 2012-2015 and 2016-2020 periods (Figure 2).

**Figure 3.**
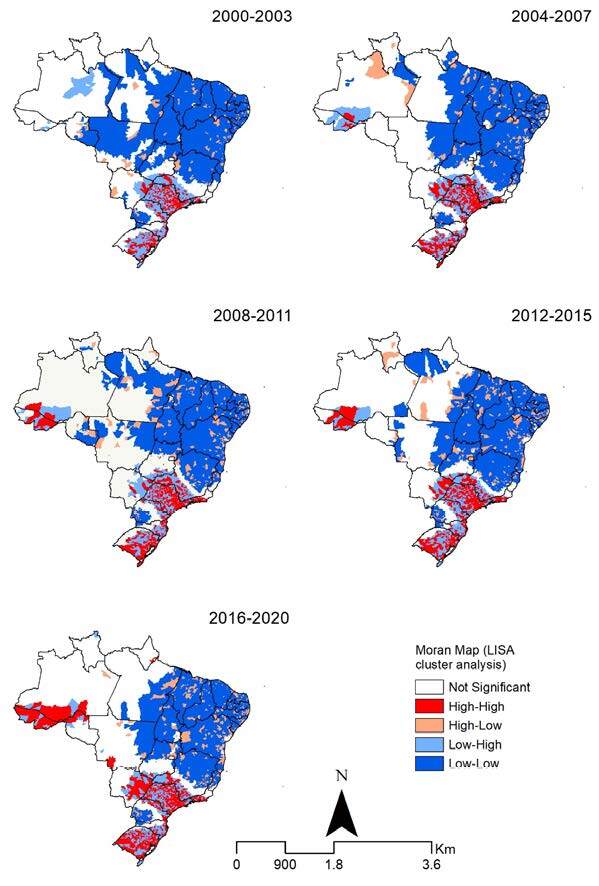
Spatial and spatiotemporal LISA cluster analysis (Moran map) of Hepatitis C-related mortality rates (per 100,000 inhabitants) by the municipality of residence in Brazil, 2000–2020

Spatial autocorrelation identified clusters of municipalities with high rates surrounded by municipalities with high rates (high-high) in the five analyzed time periods (Moran’s I 2000–2003: 0.196199 [Z-score 57.631538, p-value < 0.0001], 2004–2007: 0.200011 [Z-score 58.713194, p-value < 0.0001], 2008–2011: 0.195546 [Z-score 57.399518, p-value < 0.0001], 2012–2015: 0.202017 [Z-score 59.227519, p-value < 0.0001], 2016–2020: 0.122914 [Z-score 36.179333, p-value < 0.0001]). Municipalities with high rates surrounded by municipalities with high rates (persistent over the five analyzed periods) (high-high) were identified in the states of São Paulo, south of Rio de Janeiro, and Rio Grande do Sul. After 2004, municipalities with a concentration of high rates surrounded by municipalities with high rates were identified in Acre, southern Amazonas, eastern Mato Grosso do Sul, southern Minas Gerais, in the north and in the coastal region of Paraná, and in the coastal region of Santa Catarina (high-high) (Figure 3).

**Figure 4.**
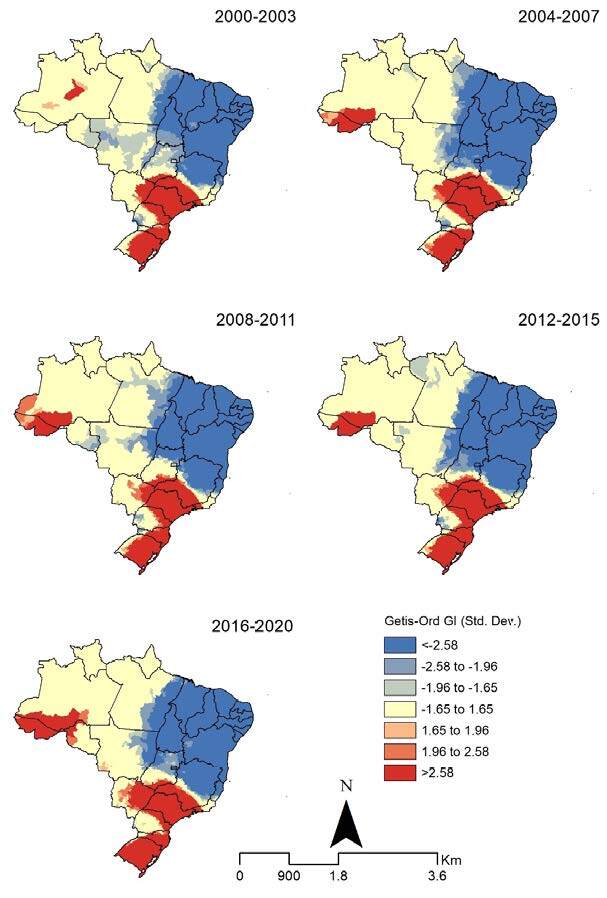
Spatial and spatiotemporal hot spot analysis (Getis–Ord Gi*) of Hepatitis C-related mortality rates (per 100,000 inhabitants) by the municipality of residence in Brazil, 2000–2020

The spatial correlation of hot areas using the Getis-Ord Gi* method revealed a concentration of agglomerations with high rates in the five analyzed periods (Getis-Ord General G 2000– 2003: 0.000002 [Z-score 25.187988, p-value < 0.0001], 2004– 2007: 0.000002 [Z-score 23.588958, p-value < 0.0001], 2008-2011: 0.000002 [Z-score 21.860611, p-value < 0.0001], 2012-2015: 0.000002 [Z-score 20.295399, p-value < 0.0001], 2016-2020: 0.000001 [Z-score 7.604160, p-value < 0.0001]). Areas with high rates in the five analyzed periods were identified in the states of São Paulo, southern Minas Gerais, southern Rio de Janeiro, northern Paraná, southern and in the coastal region of Santa Catarina, eastern of Mato Grosso do Sul, and southern Rio Grande do Sul. The states of Acre and southern Amazonas showed concentrations of high rates after 2004, which spread to northern Rondônia in the period 2016-2020 (Figure 4).

## DISCUSSION

The World Health Organization (WHO) proposed to reduce new hepatotropic virus infections and their associated mortality by 90% and 65%, respectively, by 2030^20^.

In agreement with WHO’s proposal, the Brazilian Ministry of Health outlined a national strategy to achieve this goal. The overall objective of the Plan for the Elimination of Hepatitis C in Brazil is to expand access to prevention, diagnosis, and treatment of hepatitis C, involving the three levels of government (federal, state, and municipal), to reduce new infections and mortality^21^.

A previous study evaluated hepatitis C notification and mortality in Brazil from 2008 to 2018. Regarding hepatitis C reporting, a temporal pattern of increase was observed in ten states after implementing the change in the mandatory reporting process: three states in the North region from 2008 (Rondônia, Amazonas, and Pará); five states in the Northeast region (Piauí, Ceará, and Bahia, starting in 2008, Alagoas from 2013, and Rio Grande do Norte from 2015); one state in the Southeast region starting in 2008 (Minas Gerais), and also one in the South region from 2008 (Paraná). On the other hand, decreasing trends were observed in four states: three states in the North region from the time series (Acre, Roraima, and Amapá) and one state in the South region from 2013 (Santa Catarina)^6^.

Temporal trends in mortality from viral hepatitis were updated with concordant results. The temporal trend of hepatitis A, B, other viral hepatitis, and unspecified hepatitis decreased in Brazil, while mortality from chronic hepatitis increased in the North and Northeast regions.

Hepatitis C was included in 0.26% of the DC in Brazil from 2000 to 2020. Fulminant hepatitis, the fatal form of acute hepatitis, is extremely rare and would not justify such a large number of deaths. The large number of deaths associated with acute hepatitis may reflect cases of acute to chronic liver failure.

When analyzing mortality trends over time, we observed a progressive increase in mortality until 2016, with a significant decrease from 2016 to 2020. This fact is probably related to the introduction of direct-acting antiviral (DAA) treatments in Brazil in 2015. In Europe, the number of liver transplants (LT) for Hepatitis C viral infection (HCV) is declining rapidly for both HCV cirrhosis and HCV hepatocellular carcinoma indications^6^. The predominance of deaths in men with higher adjusted mortality in the 60–69 age group reflects the epidemiological profile of infected patients in Brazil. Age-adjusted mortality was almost twice as high in men than in women, which may be explained by the association of overlapping risk behaviors such as alcoholism.

Age-adjusted mortality was higher in those over 60 years of age, which may be explained by the natural history of hepatitis C. It tends to increase severity later in the course of the infection and the later the contamination occurs.

The highest mortality from hepatitis C occurring after 60 years of age is explained by the fact that most of patients with hepatitis C in Brazil became infected before 1980 and the emergence of HIV, which introduced the implementation of standard precautions against the transmission of blood borne infections and the screening in blood banks in 1993^3^.

The regional distribution of hepatitis C mortality in Brazil is quite heterogeneous, with more than twice as many deaths in the South and Southeast regions as in the Central-West, Northeast, and North regions. Although mortality is still significant in the South, it is higher in the North, Central-West, and Northeast regions, in descending order. This may reflect better access to diagnosis, albeit late, and less access to direct-acting antiviral (DAA) treatment. The temporal and spatial maps show a high and increasing mortality in the state of Acre, in the far north of Brazil. A recognized high prevalence of infection in its population, together with delayed diagnosis and/or less access to new treatments, could be possible explanations for this finding^22^.

The higher adjusted mortality for White people is explained by their predominance in the southern and south-eastern Brazil, where most deaths are concentrated. In the temporal analysis, there is a significant decrease in mortality in Asians and White. On the other hand, there is a significant increase in mortality among Black and *Pardo* people, which may be explained by the difficulty this population has of accessing new treatments.

Deaths predominated among people living in the capital cities compared with those living in the countryside. However, there is a clear temporal trend in the reduction of mortality in the capital cities and a significant increase in mortality in residents of the countryside. The predominance of hepatitis C deaths in municipalities with low and very low social vulnerability index (IVS) can be explained by the better provision of testing and diagnosis. Similarly, the significant increase in mortality in municipalities with high and very high IVS can be explained by the difficulty of access to diagnosis and treatment.

The spatial distribution showed a higher concentration of deaths in the states of São Paulo, Rio de Janeiro, Rio Grande do Sul, Acre, Amazonas, Pará, Rondônia, and Mato Grosso do Sul, with several clusters identified. In 2020, the ranking of capitals with the highest hepatitis C detection rates showed nine capitals with rates higher than the national rate of 4.4 cases per 100,000 inhabitants: Porto Alegre-RS (47.2 cases per 100,000 inhabitants) with the highest rate among the capitals, followed by Curitiba-PR (11.9), Boa Vista-RR (11.0), São Paulo-SP (9.6), Rio Branco-AC (8.5), Florianópolis-SC (8.3), Cuiabá-MT (6.0), Manaus-AM (5.4), and Porto Velho-RO (5.0). The coincidence of the mortality clusters in several capital cities with high mortality and the increase in the number of diagnoses/notifications suggests that these regions may be testing more and therefore, the DC for hepatitis C are higher. This interpretation highlights the increase in mortality in the state of Acre and in the south of the Amazon.

Despite being as socially vulnerable as the rest of the region, the state of Acre may have had a successful hepatitis C testing policy after 2004. The high mortality in Acre, similar to that reported in the South region, is also explained by the high prevalence of hepatitis C in the state, which is well above the national average. The municipalities of Tarauacá and Cruzeiro do Sul stand out in the state, with 7% and 3.8% of the population are infected with hepatitis C, respectively^23^.

Although it represents the largest number of deaths among hepatitis, hepatitis C has shown a decrease in the mortality coefficient and in the number of deaths since 2015, with a 25% decrease in the number of deaths in this period. It may be related to the beginning of the offer of highly effective treatments, which cure more than 95% of cases. In Brazil, 1.5–2 million people are infected with HCV, which remains the leading cause of cirrhosis and hepatocellular carcinoma in the country. More than 130,000 Brazilians have been cured from hepatitis C by the new direct-acting antiviral (DAAs) since the drugs were included into the Brazilian Unified Health System (SUS) in 2015. This represents 23% of the target to eliminate the infection in the country by 2030^24 , 25^. This study provides a baseline for new studies on mortality and supports the achievement of the projected goals for the elimination of hepatitis in Brazil by 2030.

From 2008 to 2018, 136,759 newly diagnosed cases of hepatitis C were reported considering anti-HCV and HCV-RNA positivity, and 271,624 newly diagnosed cases were reported considering one or another positive test. The majority of cases were concentrated in the Southeast (61%) and South (26.2%) regions. The joinpoint regression model showed an increasing trend in the detection rate of hepatitis C in Brazil, but there was a decreasing trend in the mortality rate during the period analyzed. It is important to highlight that the criteria for reporting a case of hepatitis C changed in 2015, which explains part of the increase in diagnosed cases. Previously, a molecular test was required to report a case of hepatitis C; after 2015, only a serological test was sufficient for reporting.

The limitations of this study are related to the databases used. Despite the progress made, there is still evidence of variation in performance across the country in terms of recording all the deaths and the quality of completion and investigation of DCs^26^. Although it is recognized that, since 2005, the Ministry of Health has initiated a process to improve the system coverage by reducing the number of deaths with undetermined causes, based on the development of new computerized tools and training and/or qualification courses for new coders, there are still challenges in terms of the quality of the records^27^.

The high mortality due to hepatitis C in Brazil has decreased heterogeneously, as shown by our results. Efforts for early diagnosis and treatment imply an important reduction in costs for the SUS. In the early and late phases, outpatient prescriptions were the largest cost component, whereas, in the terminal phase, the costs were primarily driven by inpatient care. The average annual cost per patient for hepatitis C treatment was $6,120 in the early phase and increased to $15,480 in the late phase. The substantial increase in costs highlights the role of early diagnosis and treatment in preventing hepatitis C sequelae and in reducing healthcare costs^27^.

Mortality rates among people successfully treated for hepatitis C in the era of interferon-free DDAs are highly compared in the general population. Drug- and liver-related causes of death were the main causes of excess mortality^22^.

## CONCLUSION

This study provides important data on the behavior of hepatitis C in Brazil over a 20-year period. A change in the disease epidemiology has been observed, mainly due to changes in diagnostic confirmation criteria and the introduction of new drugs, which have contributed to a reduction in mortality in recent years. Marked differences in diagnosis and mortality call the need for a differentiated elimination strategy. The data point to the need for increased diagnostic coverage in the non-capital cities and strong investment in treatment in states such as Acre, São Paulo, Rio de Janeiro, Paraná, and Rio Grande do Sul. The elimination of hepatitis C as originally proposed by the WHO seems to be a distant scenario.

## References

[1] Barré T., Bourlière M., Parlati L., Ramier C., Marcellin F., C Protopopescu (2024). Hepatitis C virus cure from direct-acting antivirals and mortality: Are people with and without a history of injection drug use in the same boat? (ANRS CO22 Hepather cohort). Drug Alcohol Review.

[2] Pereira L. M. M. B., Martelli C. M. T., Moreira R. C., Merchan-Hamman E., Stein A. T., RMA Cardoso (2013). Prevalence and risk factors of Hepatitis C virus infection in Brazil, 2005 through 2009: A cross-sectional study. BMC Infect Dis.

[3] Ministério da Saúde (BR) (2023). Boletim Epidemiológico - Hepatites Virais 2023 Departamento de HIV/Aids, Tuberculose, Hepatites Virais e Infecções Sexualmente Transmissíveis. Secretaria de Vigilância em Saúde e Ambiente [Internet].

[4] Pawlotsky J. M., Negro F., Aghemo A., Berenguer M., Dalgard O., G Dusheiko (2020). EASL recommendations on treatment of hepatitis C: Final update of the series. J Hepatol.

[5] Lobato C. M. O., Codes L., Silva G. F., Souza A. F. M., Coelho H. S. M., MLA Pedroso (2019). Direct antiviral therapy for treatment of hepatitis C: A real-world study from Brazil. Annals of Hepatology.

[6] Perazzo H., Pacheco A. G., Luz P. M., Castro R., Hyde C., J Fittipaldi (2017). Age-standardized mortality rates related to viral hepatitis in Brazil. BMC Infectious Diseases.

[7] Brito R. J. V. C., Silva L. F., Santos M. B., Moura P. M. M. F., Souza C. D. F., Carmo R. F. (2022). A time series analysis of detection and mortality of hepatitis C in Brazil, 2008–2018. BMC Infec Dis.

[8] Sousa L. F. O., Santos E. R. S., Oliveira R. M., Andrade R. L. B., Batista J. F. C., Lima S. O. (2023). Hepatitis mortality in Brazil and regions, 2001–2020: temporal trend and spatial analysis. Rev bras epidemiol.

[9] Instituto Brasileiro de Geografia e Estatística (IBGE) (2023). Cidades e Estados [Internet]. Rio de Janeiro.

[10] Instituto de Pesquisa Econômica Aplicada (IPEA) (2015). Atlas da Vulnerabilidade Social nos Municípios Brasileiros [Internet].

[11] Instituto Brasileiro de Geografia e Estatística (IBGE) (2020). Características gerais dos domicílios e dos moradores 2019 – PNAD Contínua. Pesquisa Nacional por Amostra de Domicílios Contínua.

[12] Organização Mundial da Saúde (OMS) (2019). International Statistical Classification of Diseases and Related Health Problems 10th Revision. ICD-10 Version: 2019 [Internet].

[13] Ministério da Saúde (BR) (2018). Departamento de Doenças de Condições Crônicas e Infecções Sexualmente Transmissíveis.

[14] Ministério da Saúde (BR) (2021). Informações de Saúde (TABNET). DATASUS [Internet].

[15] StataCorp L. L. C. (2021). Stata 11©. College Station [Internet].

[16] Institute National Cancer (2021). Joinpoint Trend Analysis Software [Internet]. https://surveillance.cancer.gov/joinpoint.

[17] Kim H. J., Fay M. P., Feuer E. J., Midthune D. N. (2000). Permutation tests for joinpoint regression with applications to cancer rates. Statistics Med.

[18] Dunn C. E., Kingham S. P., Rowlingson B., Bhopal R. S., Cockings S., Foy C. J. W. (2001). Analysing spatially referenced public health data: A comparison of three methodological approaches. Health Place.

[19] Anselin L. (1995). Local Indicators of Spatial Association–LISA. Geogr Anal.

[20] World Health Organization (WHO) (2016). Global health sector strategy on viral hepatitis 2016–2021. Towards ending viral hepatitis [Internet].

[21] Brasil (2018). Plano para Eliminação da Hepatite C no Brasil.

[22] Hamill V., Wong S., Benselin J., Krajden M., Hayes P. C., D Mutimer (2023). Mortality rates among patients successfully treated for hepatitis C in the era of interferon-free antivirals: population based cohort study. BMJ.

[23] Dantas T. O. M. (2010). Aspectos epidemiológicos da infecção pelo vírus da hepatite C e coinfecções com os vírus B e delta no estado do Acre, Amazônia Ocidental Brasileira [Internet].

[24] Cooke G. S., Andrieux-Meyer I., Applegate T. L., Atun R., Burry Cheinquer H. (2019). Accelerating the elimination of viral hepatitis: a Lancet Gastroenterology & Hepatology Commission. Lancet Gastroenterol Hepatol.

[25] World Health Organization (WHO) (2016). Global health sector strategy on viral hepatitis 2016-2021. Global Hepatitis Programme Department of HIV/AIDS. Geneva.

[26] Diógenes V. H. D., Pinto  E. P., Gonzaga M. R., Queiroz B. L., Lima E. E. C., Costa L. C. C. (2022). Differentials in death count records by databases in Brazil in 2010. Rev Saúde Pública.

[27] Erman A., Sahakyan Y., Everett K., Greenaway C., Janjua N., JC Kwong (2024). Hepatitis C Attributable Healthcare Costs and Mortality among Immigrants: A Population-Based Matched Cohort Study. Can J Gastroenterol Hepatol.

